# Psychometric Properties of the Behavioral–Emotional Regulation Questionnaire in Peruvian Adults (BERQ-PA)

**DOI:** 10.3390/bs15020224

**Published:** 2025-02-16

**Authors:** Dennis López-Odar, Marivel Aguirre-Morales, Ingrid Cirilo-Acero, Edmundo Hervias-Guerra, Arístides Vara-Horna, Sabina Deza-Villanueva

**Affiliations:** 1 Facultad de Psicología, Universidad Nacional Federico Villarreal, Lima 15082, Peru; maguirre@unfv.edu.pe (M.A.-M.); icirilo@unfv.edu.pe (I.C.-A.); ehervias@unfv.edu.pe (E.H.-G.); 2 Facultad de Ciencias Administrativas y Recursos Humanos, Universidad de San Martín de Porres, Lima 15009, Peru; avarah@usmp.pe; 3 Facultad de Psicología y Humanidades, Universidad Femenina del Sagrado Corazón, Lima 15012, Peru; sdezav@unife.edu.pe

**Keywords:** validation, emotional regulation, behavioral–emotional regulation, factorial invariance

## Abstract

Behavioral and cognitive regulation of emotions is crucial for adaptation and mental health. Measuring it with valid and reliable instruments is essential, especially in Peru. The objective of the study was to evaluate the psychometric properties of the Peruvian version of the Behavioral Regulation of Emotions Questionnaire (BERQ-PA) in a sample of 403 adults from Lima, aged between 18 and 59 years (M = 32.3, SD = 10.1; 65.8% women). Participants completed the BERQ-PA, the Emotion Regulation Questionnaire (ERQ), and the General Health Questionnaire (GHQ-28). Confirmatory factor analysis (CFA) was used to assess the validity of the internal structure. The BERQ-PA scores were correlated with those of the ERQ and GHQ-28 to check concurrent and convergent validity. Reliability was established by internal consistency analysis, and factorial invariance according to sex was evaluated using a multigroup CFA. The CFA confirmed the original five-factor model; however, two items had factor loadings lower than 0.40. For this reason, an alternative five-factor, 18-item model was evaluated that showed optimal fit indices (*S-Bχ*^2^ = 299, *df* = 125, *χ*^2^/*df* = 2.39, CFI = 0.959, TLI = 0.950, RMSEA = 0.048 [90% CI: 0.039–0.057], SRMR = 0.069). The alpha and omega coefficients of the five subscales were greater than 0.70, confirming reliability. The correlations between the BERQ, ERQ, and GHQ-28 subscales evidenced convergent and concurrent validity. Factorial invariance according to sex was confirmed. The BERQ-PA is a valid and reliable measure of the behavioral regulation of emotions, standing out for its usefulness for research, psychological assessment, and the assessment of interventions in the Peruvian context.

## 1. Introduction

Emotional regulation involves processes through which the way positive or negative emotions are experienced and expressed is modulated and modified (Gross, 1998a). Gross and Thompson (2007) suggest that emotional regulation manifests as a process or a set of strategies deployed throughout the emotion generation cycle. In this process, each individual sets goals to assess their circumstances to regulate their emotional response (Gross, 2015). In this sense, emotional regulation influences the onset, intensity, and duration of emotions and their expression or suppression. (Garnefski et al., 2001; Gross & Thompson, 2007).

As a process, emotional regulation refers to automatic or deliberate efforts to manage our emotions (Liu & Thompson, 2017). It also encompasses a series of implicit cognitive and explicit behavioral manifestations that influence the way one responds to external demands, adapts to stressful situations, and maintains health and well-being (Garnefski et al., 2001; Compas et al., 2001; McRae et al., 2020; Fombouchet et al., 2024). Evidence suggests that individuals employ various strategies to regulate their emotions, and success in this task is associated with higher levels of motivation, functionality, and the development of adaptive behaviors in stressful contexts.

There is a general consensus that emotional regulation comprises a set of strategies. Gross (1998b) identifies two main groups: antecedent-focused strategies, which impact the occurrence of emotions (such as situation selection and modification, attention deployment, and cognitive change), and response-focused strategies, which contribute to the modulation of emotions. On the other hand, Garnefski et al. (2001) argue that emotional regulation involves biological, social, behavioral, and cognitive components that depend on individual characteristics and context.

Furthermore, the literature reports evidence on how emotional regulation strategies are associated with maladaptive responses and psychological disorders (Aldao et al., 2010; Mennin et al., 2007; Gross & Jazaieri, 2014). Adaptive behaviors are promoted when individuals use effective strategies to regulate negative emotions; however, when there are difficulties in emotional regulation, especially intense emotions in stressful situations, the likelihood of psychological disorders such as depression, anxiety, suicidal behavior, and other mental health problems increases (Liu & Thompson, 2017; Lopez & Denny, 2019; McMahon et al., 2019; Stikkelbroek et al., 2016; Wingo et al., 2015).

Given the role of emotional regulation in understanding and explaining well-being and mental health issues, instruments have been designed to evaluate it validly and reliably. The literature review has shown that self-report scales predominate, highlighting the extensive use of the Emotion Regulation Questionnaire (ERQ), which evaluates cognitive reappraisal and suppression strategies (Gross & John, 1998), and the Difficulties in Emotion Regulation Scale (DERS), designed by Gratz and Roemer (2004). Both instruments explore emotion regulation strategies without distinguishing between cognitions and behaviors. This constitutes a limitation, as Garnefski et al. (2001) noted, because cognitive and behavioral efforts to regulate emotions possess a distinct nature.

Conceiving emotional regulation as a set of processes or strategies without differentiating how people think and act constitutes a limitation for its understanding, research, and measurement. Garnefski et al. (2002) propose an approach recognizing the existence of multiple forms or strategies of emotional regulation, some linked to cognitive processes and others to behavioral processes. Under this approach, people facing a situation and regulating their emotions can use cognitive strategies such as positive focus or blaming others (Garnefski et al., 2002). They can also use behavioral strategies such as Seeking Distraction or Seeking Social Support (Kraaij & Garnefski, 2019).

Cognitive strategies refer to thoughts or mental processes aimed at modifying the evaluation of situations to achieve emotional regulation. In contrast, behavioral strategies involve physical actions or activities that individuals perform to control their emotions (Garnefski et al., 2001). Distinguishing cognitive and behavioral components in emotional regulation processes has allowed for a better understanding of their linkage to health and the development of psychological disorders (Tuna, 2021). Moreover, this segmentation has practical implications for measuring emotional regulation and designing psychological interventions (Garnefski & Kraaij, 2007; Kraaij & Garnefski, 2019).

Considering this approach, Garnefski et al. (2001) propose that measuring emotional regulation should involve independently assessing cognitive and behavioral dimensions. For this reason, they designed the Cognitive Emotion Regulation Questionnaire [CERQ] (Garnefski & Kraaij, 2007; Garnefski et al., 2002), which explores nine cognitive emotion regulation strategies and has been widely used in research and clinical practice (Domínguez-Lara & Medrano, 2016; Zhu et al., 2008; Xiao et al., 2011).

Kraaij and Garnefski (2019) designed the Behavioral Emotion Regulation Questionnaire (BERQ) to assess five behavioral strategies commonly used to regulate emotions arising from stressful situations to enhance contributions to the measurement of emotional regulation. The development of the BERQ involved multiple pilot studies. The initial version comprised 32 items to measure dispositional coping (behavioral styles in response to threatening or stressful situations) and situational coping (behavioral strategies used in specific stressful situations).

In its first version, the BERQ evaluates behavioral emotion regulation strategies in individuals aged 12 and older, including adults. Additionally, it is organized into five subscales of four items each:

(a) Seeking Distraction: Involves coping with a stressful event by engaging in activities that divert attention from experienced emotions.
(b) Withdrawal: Refers to distancing oneself from situations and avoiding interaction with involved individuals.
(c) Actively Approaching: Engaging in behaviors to address stressful events directly.
(d) Seeking Social Support: Behaviors through which emotions are expressed and support from others are sought to cope with the stressful event.
(e) Ignoring: Responding to a stressful event as if nothing had happened.

Kraaij and Garnefski (2019) evaluated the psychometric properties of the BERQ with a sample of 457 adults in the Netherlands. This generated valid evidence based on the internal structure obtained through principal component analysis (PCA). This procedure determined the number of factors using the Kaiser rule and oblimin rotation. The results confirmed a five-dimensional structure: Seeking Distraction, Ignoring, Withdrawal, Actively Approaching, and Seeking Social Support.

Regarding criterion validity, behavioral strategies were significantly related to depressive symptoms. Seeking Distraction, Actively Approaching, and Seeking Social Support strategies were negatively correlated with depression scores, while Withdrawal and Ignoring strategies were positively associated with depressive symptoms. Additionally, significant correlations were established between anxiety and all BERQ subscales, except the Seeking Distraction strategy (Kraaij & Garnefski, 2019). Finally, internal consistency and score stability were verified using Cronbach’s Alpha coefficient, with values ranging from 0.86 to 0.93, and the test–retest method, with Pearson correlation coefficients ranging from 0.47 to 0.75.

Subsequently, versions and adaptations of the BERQ have been developed in various countries. In a study conducted in Romania by Ursu et al. (2024), a confirmatory factor analysis (CFA) was applied to a sample of 399 participants aged between 18 and 67. The results demonstrated a good model fit (*χ*^2^ (141) = 311.72, *p* < 0.001), supported by satisfactory fit indices: CFI = 0.93, TLI = 0.91, SRMR = 0.06, and RMSEA = 0.06. Additionally, evidence of concurrent validity showed inverse correlations between the BERQ subscales, and the scores obtained on the Patient Health Questionnaire for Depression and Anxiety (PHQ–4), the Perceived Stress Scale Short Form (PSS), and the Positive and Negative Affect Schedule (PANAS). The instrument’s reliability was high for each factor, with Alpha (*α*) and Omega (*ω*) coefficients ranging from 0.77 to 0.86. Likewise, temporal stability measures were obtained based on intraclass correlation coefficients, ranging from 0.64 to 0.90.

Similarly, Abdollahpour-Ranjbar et al. (2023) conducted a study to evaluate the psychometric properties of a version of the BERQ adapted to the Iranian context (BERQ-PV). The study included 556 adults from the general population and 92 patients diagnosed with anxiety or depression. After performing a confirmatory factor analysis (CFA), the results showed an acceptable fit for the five-subscale model (*χ*^2^ = 384.61, *p* < 0.05, CFI = 0.91, NNFI = 0.89, SRMR = 0.06, and RMSEA = 0.05). Concurrent validity was demonstrated through significant correlations between the BERQ-PV subscales and those of the Depression, Anxiety, and Stress Scale (DASS-21). Moreover, significant correlations between most BERQ-PV subscales and the CERQ evidenced convergent validity. Finally, the instrument demonstrated adequate internal consistency according to the criteria proposed by Hair et al. (2010), with Cronbach’s alpha coefficients ranging from 0.59 to 0.78. Additionally, temporal stability was evidenced using the test–retest method, yielding intraclass correlation coefficients ranging from 0.63 to 0.82.

In China, Zhao et al. (2020) also evaluated the psychometric properties of an adaptation of the BERQ. A total of 816 university students participated (38.6% were male and 61.4% were female), ranging from 17 to 21 years. They generated evidence of validity by applying CFA. The results showed an acceptable fit for the five-factor model (*S-Bχ*^2^ = 469.11, *df* = 160, CFI = 0.937, TLI = 0.925, RMSEA = 0.049, SRMR = 0.052). Reliability metrics were reported using the internal consistency method, with Alpha coefficients (*α* = 0.71–0.85) and Spearman–Brown split-half coefficients (0.66–0.85). Additionally, the test–retest temporal stability method yielded acceptable values in the intraclass correlation coefficients (0.55–0.67).

Similarly, Tuna (2021) conducted a study in Türkiye with the participation of 320 adults aged between 18 and 50 years. The objective was to determine the psychometric properties of the BERQ. A cross-validation analysis compared estimates obtained through Principal Component Analysis (PCA) and Confirmatory Factor Analysis (CFA). The Kaiser–Meyer–Olkin (KMO) measure and Bartlett’s sphericity test confirmed the exploratory analysis’s suitability. The results of PCA and CFA supported the internal structure of five subscales or strategies. It is important to note that a better fit was evidenced in a modified five-factor model (*S-Bχ*^2^ = 312.13, CFI = 0.87, NNFI = 0.84, RMSEA = 0.08) compared to the original model (*S-Bχ*^2^ = 322.64, CFI = 0.86, NNFI = 0.83, RMSEA = 0.08). Cronbach’s alpha coefficients ranged from 0.72 to 0.88. In contrast, omega coefficients varied between 0.70 and 0.89, supporting the internal consistency of the measurement.

In Peru, Domínguez-Lara et al. (2022) translated the BERQ and evaluated its validity and reliability in a sample of 315 university students aged between 16 and 44. Using CFA and exploratory structural equation modeling, they assessed the fit of the five-strategy model proposed in the original version of the questionnaire (Kraaij & Garnefski, 2019). Both procedures demonstrated the pentafactorial structure of the BERQ and generated acceptable fit indices. For the CFA, the obtained indices were: *χ*^2^ = 320.45 (*p* < 0.001), CFI = 0.91, RMSEA = 0.91 (90% CI: 0.83–0.99), WRMR = 1.387. Furthermore, most item loadings were greater than 0.60. As evidence of predictive validity, the regression analysis showed that the behavioral emotion regulation strategies significantly predicted distress (*R*^2^ = 0.294) and eustress (*R*^2^ = 0.288). The strategies of Seeking Distraction, Ignoring, and Withdrawal predicted distress, while Seeking Distraction, Actively Approaching, and Withdrawal predicted eustress. Additionally, they reported that the reliability of the questionnaire was adequate. Cronbach’s alpha coefficients ranged between 0.707 and 0.845, and McDonald’s Omega coefficients ranged from 0.725 to 0.864, thus reflecting the measurement’s reliability.

Considering the previously described psychometric evidence, the BERQ is a valid and reliable instrument for measuring emotional regulation. Its relevance lies in its ability to explore behavioral strategies for managing emotions during stressful events. Moreover, previous studies have shown how these strategies are associated with adaptive behaviors and symptomatology related to psychological disorders (Garnefski et al., 2017; Vizioli, 2022), thus increasing the BERQ’s importance and utility in research and clinical practice.

Although a translated version of the BERQ is available in Peru for university students (Domínguez-Lara et al., 2022), the questionnaire’s psychometric properties have not been evaluated in other age groups. Additionally, the National Center for Epidemiology, Prevention, and Control of Diseases (2023) and the Ministry of Health (2024) highlight the high prevalence of mental health problems in the Peruvian context, which demands the design of intervention programs, and the availability of instruments adapted to assess variables associated with this issue. This reality justifies the pertinence of evaluating the psychometric properties of the BERQ, particularly in the adult population.

For this reason, the present study aimed to evaluate the psychometric properties of a Peruvian version of the Behavioral Emotion Regulation Questionnaire (BERQ-PA) in a sample of Lima adults. The specific objectives of the research included generating evidence of convergent, concurrent, and internal structure validity, as well as evaluating the internal consistency of the scores and factorial invariance by sex.

Considering the study objectives, the present study hypothesizes that the confirmatory factor analysis (CFA) confirms the five-factor structure of the BERQ-PA, demonstrating an adequate model fit (Hypothesis 1). Regarding convergent validity, the more adaptive behavioral emotion regulation strategies (Seeking Distraction, Actively Approaching, and Seeking Social Support) are expected to positively correlate with cognitive reappraisal and negatively or not significantly correlate with expressive suppression (Hypothesis 2). Similarly, the less adaptive strategies (Withdrawal and Ignoring) positively correlate with expressive suppression and negatively or not significantly correlate with cognitive reappraisal (Hypothesis 3). For concurrent validity, it is hypothesized that the more adaptive strategies (Seeking Distraction, Actively Approaching, and Seeking Social Support) negatively correlate or are not significantly associated with somatic symptoms, anxiety, social dysfunction, and depression (Hypothesis 4). Conversely, the less adaptive strategies (Withdrawal and Ignoring) positively correlate with somatic symptoms, anxiety, social dysfunction, and depression (Hypothesis 5). Additionally, it is predicted that the BERQ-PA demonstrates adequate internal consistency, as indicated by satisfactory Cronbach’s alpha and McDonald’s omega coefficients for all subscales (Hypothesis 6). Finally, it is expected that factorial invariance across sex is confirmed, indicating that the measurement model functions equivalently for men and women (Hypothesis 7).

## 2. Materials and Methods

### 2.1. Participants

The sample consisted of 403 adults from Metropolitan Lima who completed an online questionnaire distributed through social media. Participants were selected through non-probability sampling, and the sample size was determined following the methodological criteria recommended for psychometric studies and the use of Structural Equation Models (Ruiz et al., 2010).

The sociodemographic characteristics of the sample are presented in Table 1. Of the participants, 65.8% were women, and 34.2% were men. Their ages ranged from 18 to 59 years (*M* = 32.3, *SD* = 10.1). Of the participants, 95.5% reported having a higher education level (technical or university), 70.0% reported being single, and 65.8% reported being employed. It is important to note that initially, 454 participants provided their informed consent. However, fifty-one cases were excluded for not meeting the following criteria: adults aged between 18 and 59, residing in Lima, and having Peruvian nationality.

### 2.2. Measures

*Behavioral Emotional Regulation Questionnaire (BERQ).* Designed by Kraaij and Garnefski (2019) to evaluate behavioral strategies used to cope with stressful events. The version of the BERQ translated and adapted in Peru by Domínguez-Lara et al. (2022) was used (this version can be found in the Supplementary Materials). The BERQ consists of 20 items that are grouped into five subscales or strategies: Seeking Distraction (e.g., “I set my worries aside by doing something else”), Withdrawal (e.g., “I close myself off to others”), Actively Approaching (e.g., “I do whatever is required to deal with it”), Seeking Social Support (e.g., “I share my feelings with someone”), and Ignoring (e.g., “I move on and pretend that nothing happened”). Each subscale comprises four items, answered through a 5-point Likert scale that varies from 1 (seldom) to 5 (almost always). Higher scores indicate which strategies are used more frequently.

Regarding psychometric properties, Kraaij and Garnefski (2019) reported evidence of structure-based validity through principal component analysis (PCA), confirming the structure of five subscales. They evaluated the consistency of the scores to establish reliability and reported Cronbach’s alpha coefficients that ranged from 0.86 to 0.93. They also determined the stability of the scores using the test–retest method, obtaining correlations between 0.47 and 0.75.

These validity and reliability evidence were replicated in different countries and in samples of young people and adults, confirming validity based on internal, convergent, discriminant, and predictive structure, as well as internal consistency and stability of scores (Abdollahpour-Ranjbar et al., 2023; Tuna, 2021; Zhao et al., 2020).

*Emotional Regulation Questionnaire (ERQ).* Designed by Gross and John (2003) to evaluate two strategies of emotional regulation: cognitive reappraisal (a strategy that modifies emotional responses during pregnancy) and expressive suppression (a strategy that modifies emotional expression, allowing emotions to be hidden, but without altering them). The Peruvian version of the ERQ was used (Gargurevich & Matos, 2010).

The instrument consists of 10 items answered through a 7-point Likert scale (1 = strongly disagree and 7 = strongly agree). Six items evaluate the cognitive reappraisal strategy (e.g., “When I want to feel less negative emotion, I change the way I’m thinking about the situation”), and four items evaluate the expressive suppression strategy (e.g., “I control my emotions by not expressing them”). The average score of the two emotional regulation strategies is calculated to interpret the results. High scores indicate that strategies are used more frequently.

The ERQ has been validated in various populations, including adolescents and adults from different countries. Evidence of validity based on internal structure has been reported through exploratory and confirmatory factor analysis. In addition, evidence of convergent validity has been generated by analyzing the correlations of their scores with other instruments (Cabello et al., 2013; Pagano & Vizioli, 2021; Pérez Sánchez et al., 2020; Westerlund & Santtila, 2018). In terms of reliability, ERQ scores have demonstrated temporal stability and internal consistency in both subscales, with Cronbach’s alpha and McDonald’s Omega coefficients ranging from 0.70 to 0.87 (Pagano & Vizioli, 2021; Pérez Sánchez et al., 2020).

The present study evaluated the internal consistency of the scores, obtaining the following coefficients: cognitive reappraisal, *ω* = 0.804 [95% CI = 0.773, 0.832], and suppression, *ω* = 0.775 [95% CI = 0.739, 0.810]. The total scores of the ERQ subscales were used to establish the convergent validity of the BERQ-PA.

*General Health Questionnaire (GHQ-28).* Designed by Goldberg and Hillier (1979) to assess mental health, it has been used in different populations and presents brief versions that have proven to be valid and reliable (Delgado-Gómez et al., 2013; López-Castedo & Domínguez, 2010; Molina et al., 2006). In Peru, there is an adaptation in the adult population exposed to COVID-19, conducted by Ames-Guerrero et al. (2020).

The version made up of 28 items was used, which explores somatic symptoms (items 1 to 7; e.g., “Have you felt that you were ill?”), anxiety/insomnia (items: 8 to 14; e.g., “Have you constantly felt overwhelmed or under stress?” and “Have you had difficulty sleeping all night?”), social dysfunction (items: 15 to 21; e.g., “Have you been satisfied with the way you do things?”), and depression (items 22 to 28; e.g., “Do you feel that life is not worth living?”). Each item is scored on a 4-point Likert scale to determine the severity of symptoms over the past few weeks. Response scores range from 0 (not at all) to 3 (much more than usual).

The psychometric evidence that has been generated corresponds to the validity of content, criterion, and validity based on the internal structure; a four-dimensional structure was verified (Ames-Guerrero et al., 2020; Prady et al., 2013; Pérez-Moreno et al., 2010; Vergara-Moragues & González-Saiz, 2020; Vallejo et al., 2014; Willmott et al., 2008). In addition, the internal consistency of the subscale scores has been verified using Cronbach’s Alpha and McDonald’s Omega coefficients. The coefficients reported in the studies were greater than 0.70. In the present study, the internal consistency of the subscales was evaluated by obtaining the following coefficients: somatic symptoms, *ω* = 0.804 [95% CI = 0.775, 0.833]; anxiety, *ω* = 0.905 [95% CI = 0.891, 0.919]; social dysfunction, *ω* = 0.800 [95% CI = 0.792, 0.823]; and depression, *ω* = 0.874 [95% CI = 0.855, 0.893].

### 2.3. Procedure

The data were collected using a virtual form created in Google Forms and administered online during the second half of 2023. The form consisted of an initial section providing information about the study and the mode of participation: voluntary, anonymous, and without incentives or economic remuneration. After this section, participants gave their statement of informed consent.

The form also included a section for collecting sociodemographic data and subsections incorporating the Behavioral Emotion Regulation Questionnaire (BERQ), translated and adapted by Domínguez-Lara et al. (2022), the Emotion Regulation Questionnaire (ERQ), and the General Health Questionnaire (GHQ-28). The collected data were subjected to quality control, filtering, and adaptation process for subsequent analysis.

Regarding the quality control and filtering of the collected information, all participants who met the established age range were considered. The design of the virtual form allowed for the complete development of questionnaires. Therefore, no lost data were reported, and it was unnecessary to apply imputation procedures.

The study was developed as part of the Research Group in Psychological Evaluation of the Faculty of Psychology, belonging to the Federico Villarreal National University. The study was approved by the Ethics Committee of the same faculty on 14 March 2024. It is important to mention that all procedures followed the ethical guidelines of the Declaration of Helsinki issued by the World Medical Association (2024) and the guidelines established in The Standards for Educational and Psychological Testing (American Educational Research Association, 2014).

### 2.4. Data Analyses

Initially, a descriptive analysis of the items was performed by calculating the mean, standard deviation, kurtosis, and asymmetry. In addition, the Mardia Test was used to assess multivariate normality. A significant result in this test shows that the data deviate from a normal distribution (Mardia, 1970). On the other hand, the homogeneity coefficients were estimated. Following the recommendations of Streiner et al. (2014), those items that showed a corrected item–test correlation coefficient of less than 0.30 should not be retained.

Evidence of validity based on the internal structure of the BERQ-PA was obtained through confirmatory factor analysis (CFA) and structural equation modeling (SEM). These statistical techniques were selected considering that, in the present study, the five-factor model and the relationships between the elements in a new sample were evaluated. In addition, they were chosen because they have been used in previous studies to assess the factor structure of BERQ (Abdollahpour-Ranjbar et al., 2023; Domínguez-Lara et al., 2022; Tuna, 2021; Kraaij & Garnefski, 2019; Zhao et al., 2020).

Due to the results of the analysis of the items’ multivariate normality and the data’s ordinal nature, the estimation method used was the Weighted Least Squares Mean and Variance Adjusted (WLSMV). This estimator is used because it is useful for analyzing ordinal data and relevant to violations of the items’ multivariate normality (Kline, 2016; Muthén & Muthén, 2017; Thompson, 2004).

Four measurement models were evaluated. The first model (M1) corresponded to the internal structure of five correlated factors proposed in the original version of the questionnaire and tested in previous studies (Abdollahpour-Ranjbar et al., 2023; Domínguez-Lara et al., 2022; Kraaij & Garnefski, 2019; Tuna, 2021; Ursu et al., 2024; Zhao et al., 2020). The second model (M2) maintained the same five-factor structure but without items 1 and 4, which presented factor loads ≥ 0.40 (Brown, 2015; Hair et al., 2010).

To evaluate a possible two-factor structure based on the classification of adaptive strategies (Seeking Distraction, Actively Approaching, and Seeking Social Support) and less adaptive strategies (Withdrawal and Ignoring), a third model (M3) was analyzed, which included the 20 items of the original version, and a fourth model (M4) considered the 18 items of the final version of the BERQ-PA.

The fit indices to evaluate the models were those proposed by Brown (2015), Hu and Bentler (1999), and Kline (2016). The Satorra–Bentler scaled chi-square statistic (*S-Bχ*^2^, *p* > 0.05), the *χ*^2^/*df* ratio (<3), the standardized root mean square residual (SRMR ≤ 0.80), and the root mean square error of approximation (RMSEA ≤ 0.08) were used as absolute fit indices. As incremental fit indices, the Comparative Fit Index (CFI ≥ 0.90 or 0.95) and the Tucker–Lewis Index (TLI ≥ 0.90 or 0.95) were used. The Expected Cross-Validation Index (ECVI) was used as a parsimony fit index to compare the models and determine the one with the greatest fit (Hu & Bentler, 1999; Schreiber et al., 2006). Considering this index, models with lower ECVI coefficients are the ones that show a better fit.

With the model that showed the greatest fit and parsimony (Model 2), multigroup confirmatory factor analysis was performed to evaluate the factorial invariance in the samples of women and men. First, the configurational invariance was assessed to check if each factor was associated with the same items in both samples. Metric invariance was then evaluated, restricting the factor loads of the items. Subsequently, the scalar invariance was verified, restricting the intercepts and assuming the equality of the sample factor loads. Finally, residual invariance was evaluated by applying loads, intercepts, and residual restrictions.

It is important to mention that the criterion for establishing invariance was to verify that the fit indices of the model did not vary significantly after the restrictions were applied (Chen, 2007; Cheung & Rensvold, 2002; Rutkowski & Svetina, 2014). As proposed by the cited authors, invariance is confirmed when: (a) the insignificance of the change in the *χ*^2^ coefficient of the hierarchically nested models (Δ*χ*^2^) is observed, (b) the change in the comparative fit indices (ΔCFI ≤ 0.01), (c) the variation in the RMSEA of the models (ΔRMSEA < 0.015), and (d) the variation in the SRMR coefficients (ΔSRMR < 0.03).

Evidence of concurrent and convergent validity was also generated. The correlations of the BERQ-PA subscales with the GHQ-28 and ERQ subscales were analyzed by calculating Pearson’s correlation coefficients. In addition, multiple regression was used to identify the BERQ subscales that explain the appearance of somatic symptoms, anxiety, depression, and social dysfunction (GHQ-28). Next, the reliability of the five subscales was estimated by evaluating the internal consistency of the scores through the ordinal Cronbach’s alpha and ordinal McDonald’s Omega coefficients. Those coefficients with values greater than or equal to 0.70 were considered acceptable (Hair et al., 2010). Descriptive and psychometric analyses were performed with the Statistical Package for Social Sciences (SPSS) version 26.0 and the following open-source statistical software: JASP version 0.17.3 (Goss-Sampson, 2018) and Jamovi 2.5.6 (The Jamovi Project, 2024).

## 3. Results

### 3.1. Preliminary Item Analysis

Table 2 presents the item analysis, focusing on estimating the mean, standard deviation, skewness, and kurtosis. Additionally, the univariate and multivariate normality of the data were assessed. The estimated skewness and kurtosis coefficients indicated that the items of the BERQ-PA have a distribution that approximates univariate normality (Forero et al., 2009). However, Mardia’s test showed that multivariate normality was not achieved (skewness = 61.90 [χ^2^ _(1540)_ = 4158.146, *p* < 0.001] and kurtosis = 569.499 [z = 43.818, *p* < 0.001]).

Additionally, the corrected item–total correlation was calculated to assess the homogeneity of the items. The coefficients obtained ranged from 0.355 (item 4) to 0.697 (item 17), demonstrating that all items are good indicators of the behavioral emotion regulation strategies measured by the questionnaire (rit > 0.30).

### 3.2. Validity Related to the Internal Structure

The original five-factor model (Kraaij & Garnefski, 2019) was initially evaluated using Confirmatory Factor Analysis. Considering the ordinal nature of the data and the non-compliance with multivariate normality, the Weighted Least Squares Mean and Variance adjusted (WLSMV) estimation method was used. As can be seen in Table 3, the original model (M1) presented adequate fit indices (*S-Bχ*^2^ = 366, *df* = 160, *χ*^2^*/df* = 2.29, CFI = 0.947, TLI = 0.937, RMSEA = 0.50, SRMR = 0.071). However, the factor loads of items 1 and 4 were less than 0.40.

For this reason, an alternative model was evaluated, consisting of the remaining 18 items grouped into the same five factors (M2). This second model showed optimal fit indices: *S-Bχ*^2^ = 299, *df* = 125, *χ*^2^/*df* = 2.39, CFI = 0.959, TLI = 0.950, RMSEA = 0.048, SRMR = 0.069. This second model showed optimal fit indices: *S-Bχ*^2^ = 299, df = 125, *χ*^2^/*df* = 2.39, CFI = 0.959, TLI = 0.950, RMSEA = 0.048, SRMR = 0.069, which were significantly different from those of the original model (*∆χ*^2^ = 67, *∆df* = 35, *p* < 0.05). In addition, all items in the different subscales presented significant factor loads ranging from 0.518 to 0.814 (see Table 4).

The Expected Cross-Validation Index (ECVI) was used to compare the models analyzed. Considering that the lowest values are associated with a best-fit model, Model 2 (ECVI = 0.829) has greater data fit, parsimony, and generalization capacity than Model 1 (ECVI = 1.047).

Finally, confirmatory factor analysis was applied to verify a bifactorial structure of more adaptive strategies (Distraction, Actively Approaching, and Seeking Social Support) and less adaptive (Withdrawal and Ignoring). Two models were evaluated (see Table 3); in the first model, the original 20 items were included, and in the second, the 18 items of the model showed a greater fit (M2). Considering the indices obtained, both models showed an inadequate fit.

### 3.3. Reliability

As in previous studies, Cronbach’s alpha and McDonald’s omega coefficients were estimated. However, due to the ordinal nature of the items, the ordinal alpha and omega coefficients were also obtained for each BERQ-PA subscale. All coefficients obtained for the different behavioral strategies exceeded the 0.70 threshold (see Table 4). In the case of ordinal alpha, values ranged from 0.754 (Seeking Distraction) to 0.830 (Actively Approaching), while the ordinal omega coefficients ranged from 0.775 (Seeking Distraction) to 0.840 (Actively Approaching). Overall, the results revealed adequate internal consistency in the scores generated by the BERQ-PA.

### 3.4. Correlation Between the BERQ-PA Subscales

Table 4 presents the correlations among the BERQ-PA subscales. Most of the correlation coefficients showed significant relationships of small or medium size among the strategies. The correlations between Seeking Social Support and Actively Approaching (*r* = 0.596, *p* < 0.001), as well as between Ignoring and Withdrawal (*r* = 0.587, *p* < 0.001), were significant, direct, and large in size. In contrast, the correlation of the strategy Seeking Social Support with the strategies Ignoring the problem and Withdrawal was not significant (*p* > 0.05).

Considering the mean, standard deviation, and the number of items in the different subscales, it was established that the strategies of Actively Approaching (*M* = 14.3, *SD* = 3.1) and Seeking Distraction (*M* = 10.8, *SD* = 2.3) are the most used by the adults in the sample. The least used strategy was Ignoring (*M* = 9.9, *SD* = 3.2).

### 3.5. Concurrent and Convergent Validity

Concurrent validity was evaluated by estimating the correlations between the BERQ-PA subscales and the GHQ-28 dimensions (see Table 5). In this context, Seeking Distraction showed significant and negative correlations with somatic symptoms, social dysfunction, depression, and anxiety (considering the partial correlation coefficient). The Withdrawal strategy showed positive correlations with all GHQ-28 dimensions. Actively Approaching only showed a significant negative correlation with social dysfunction and depression. Similarly, Seeking Social Support was negatively and significantly correlated with social dysfunction and positively correlated with anxiety (considering the partial correlation). Finally, Ignoring showed positive and significant correlations with anxiety, depression, and somatic symptoms.

It is important to mention that all significant correlation coefficients were of small size, except for the correlation between Ignoring and somatic symptoms, which was of very small size (r < 0.10). Additionally, when controlling for other subscales, it was observed that the partial correlations between the BERQ-PA strategies and the GHQ-28 dimensions were consistent with the Pearson correlation coefficients obtained.

Additional evidence of concurrent validity was obtained through multiple linear regression analysis. The results indicate that behavioral–emotional regulation strategies explain anxiety (*R*^2^ = 0.053, *F* = 12.232, *p* < 0.001), social dysfunction (*R*^2^ = 0.106, *F* = 16.816, *p* < 0.001), depression (*R*^2^ = 0.115, *F* = 18.359, *p* < 0.001), and somatic symptoms (*R*^2^ = 0.055, *F* = 12.610, *p* < 0.001).

In the case of somatic symptoms, Seeking Distraction (*β* = −0.142, *p* = 0.004) and Withdrawal (*β* = 0.218, *p* < 0.001) strategies emerge as significant explanatory factors. With regard to depression, the strategies of Seeking Distraction (*β* = −0.210, *p* < 0.001), Withdrawal (*β* = 0.211, *p* < 0.001), and Ignoring (*β* = −0.190, *p* = 0.001) stand out as explanatory factors. Likewise, Actively Approaching (*β* = −0.158, *p* = 0.002), Withdrawal (*β* = 0.198, *p* < 0.001), and Seeking Distraction (β = −0.174, *p* = 0.001) have a significant association with social dysfunction, while anxiety is related to Withdrawal (*β* = 0.226, *p* < 0.001) and Seeking Distraction (*β* = −0.118, *p* = 0.016) strategies.

Regarding convergent validity, the association between the strategies evaluated by the BERQ-PA and the ERQ subscales was analyzed (Gross & John, 2003). As expected, the strategies Seeking Distraction, Actively Approaching, and Seeking Social Support were positively and significantly correlated with cognitive reappraisal (see Table 6). On the contrary, cognitive reappraisal showed a negative correlation with Withdrawal (*r* = −0.136, *p* < 0.01) and not significant with Ignoring (*r* = 0.054, *p* < 0.05).

On the other hand, the expressive suppression subscale was positively correlated with the Withdrawal and Ignoring strategies, and negatively with the Actively Approaching and Seeking Social Support strategies. No significant correlation was found between Seeking Distraction and Expressive Suppression (*r* = 0.040, *p* < 0.05).

### 3.6. Evaluation of Factor Invariance in the Subsamples of Men and Women

The multigroup analysis performed to evaluate factor invariance demonstrated the equivalence of the measurement models in the samples of men and women (see Table 7). The results showed non-significant differences when evaluating invariance at the configurational and metric levels. The configurational invariance model (MI 1) showed an optimal fit (CFI > 0.95, RMSEA < 0.50, SMRM < 0.80) and the factor loads in each factor did not differ between the groups. For this reason, it was found that the five factors are associated with the same items in both subsamples.

By restricting the factor loads to corroborate the metric invariance (MI 2), it was evidenced that the variations in the CFI, RMSEA, and SRMR adjustment indices were less than the established cut-off points (ΔCFI ≤ 0.01, ΔRMSEA < 0.015, ΔSRMR < 0.03), accepting metric invariance (Chen, 2007). These findings were also obtained by restricting factor loads and intercepts, proving scalar invariance (MI 3).

The evaluation of the models culminated with the verification of the residual invariance by applying restrictions on loads, intercepts, and residues (MI 4). Fit ratios at this level were also optimal (CFI = 0.985, TLI = 0.985, RMSEA = 0.027, SRMR = 0.077). As in previous models, the variation in the chi-square coefficient (Δ*χ*^2^_A-B_) was not significant. In addition, the variation observed in the adjustment indices presented magnitudes that allowed strict invariance to be established.

## 4. Discussion

The current study presents compelling evidence that supports the validity and reliability of the adaptation of the Peruvian Behavioral Emotion Regulation Questionnaire (BERQ-PA). The results confirm the five-factor structure of the instrument, demonstrate its internal consistency, and establish its factorial invariance across sexes. Furthermore, the BERQ-PA displays robust convergent and concurrent validity, thereby reinforcing its utility for assessing behavioral emotion regulation within the Peruvian adult population.

### 4.1. Factor Structure and Measurement Invariance

The findings affirm that the BERQ-PA maintains the five-factor structure initially proposed by Kraaij and Garnefski (2019), thereby substantiating the presence of Seeking Distraction, Withdrawal, Actively Approaching, Seeking Social Support, and Ignoring as distinct behavioral strategies for emotion regulation. The model fit was deemed adequate following the refinement of the instrument through the elimination of items with low factor loadings, a practice that is consistent with previous validation studies (Domínguez-Lara et al., 2022; Ursu et al., 2024).

In addition to confirming the internal structure, this study also establishes factorial invariance across sexes, indicating that the BERQ-PA measures the same constructs equivalently in both men and women. This finding is crucial, as it ensures that any observed differences between groups reflect genuine variations in emotion regulation behaviors rather than measurement biases. Similar patterns of invariance have been reported in adaptations of the BERQ in other cultural contexts (Abdollahpour-Ranjbar et al., 2023; Zhao et al., 2020).

### 4.2. Convergent and Concurrent Validity

The BERQ-PA demonstrates strong convergent validity, as the expected relationships between behavioral strategies and cognitive emotion regulation processes have been observed. More adaptive strategies, such as Seeking Distraction, Actively Approaching, and Seeking Social Support, correlate with cognitive reappraisal, an emotion regulation strategy that promotes psychological resilience (Gross & John, 2003). In contrast, the less adaptive strategies, Withdrawal and Ignoring, are linked to expressive suppression, which is often associated with increased emotional distress and maladaptive coping (Aldao et al., 2010). These findings underscore the conceptual distinction between adaptive and maladaptive behavioral regulation strategies.

Regarding concurrent validity, the expected associations between emotion regulation behaviors and mental health indicators were confirmed. Participants who frequently engaged in more adaptive behavioral strategies reported lower levels of psychological distress, including reduced symptoms of anxiety, depression, and social dysfunction. Conversely, greater reliance on avoidance-based strategies, such as Withdrawal and Ignoring, was linked to increased emotional difficulties. These findings align with previous research demonstrating that behavioral strategies play a key role in mental health outcomes (Tuna, 2021; Kraaij & Garnefski, 2019).

### 4.3. Reliability and Internal Consistency

The BERQ-PA demonstrated high internal consistency, confirming its reliability as a measurement tool. All subscales showed strong coherence, indicating that the items within each factor effectively assess the intended behavioral strategies. These results are consistent with previous adaptations of the BERQ in different cultural contexts, where similar reliability levels have been observed (Tuna, 2021; Zhao et al., 2020; Ursu et al., 2024).

Considering the ordinal nature of the data, reliability analyses employed advanced statistical methods to ensure precise estimations. The decision to utilize polychoric correlations and McDonald’s omega coefficients facilitated a more accurate assessment of internal consistency, thereby enhancing the robustness of the instrument. These methodological selections are consistent with best practices in psychometric research (Domínguez-Lara, 2018; Graham, 2006).

### 4.4. Implications for Research and Practice

The validation of the Behavioral Emotion Regulation Questionnaire for Peruvian Assessment (BERQ-PA) within the Peruvian context carries significant practical implications. This instrument provides researchers and clinicians with a culturally adapted tool to assess behavioral emotion regulation, thereby improving its use in psychological evaluation, intervention design, and mental health research. Given the increasing recognition of emotion regulation as a crucial aspect of psychological well-being, the BERQ-PA may assist in identifying individuals at risk of maladaptive coping mechanisms and in directing interventions that promote adaptive strategies.

Moreover, the findings underscore the importance of considering cultural factors in emotion regulation research. While the five behavioral strategies appear stable across different populations, the frequency and effectiveness of these strategies may vary depending on sociocultural norms and environmental influences. Future studies should explore how contextual variables shape behavioral emotion regulation strategies in different demographic groups.

### 4.5. Limitations and Future Directions

Despite its contributions, this study has certain limitations. The sample was obtained through non-probabilistic methods, which may limit the generalizability of the findings. Future research should aim to enhance sample diversity by including participants from various socioeconomic backgrounds, age groups, and geographic regions to ensure broader applicability.

Furthermore, although the study demonstrates robust cross-sectional validity, future research endeavors should investigate the predictive validity of the Behavioral Emotion Regulation Questionnaire for Physical Activities (BERQ-PA) through longitudinal studies. Analyzing how behavioral emotion regulation strategies affect long-term mental health outcomes would yield significant insights into their stability and effectiveness over time.

Another important area for future research is the use of Exploratory Structural Equation Modeling (ESEM), which offers a more flexible evaluation of the relationships among items and factors. This approach has been successfully applied in previous adaptations of the BERQ (Domínguez-Lara et al., 2022) and has the potential to further enhance the understanding of the instrument’s structural features.

Finally, incorporating clinical diagnostic tools in future research would bolster the evidence backing the BERQ-PA’s clinical utility. While this study centered on a general population sample, evaluating its effectiveness in clinical settings could increase its relevance in psychological treatment and intervention programs.

## 5. Conclusions

The present study confirms that the BERQ-PA is a valid and reliable instrument for assessing behavioral emotion regulation strategies in Peruvian adults. Its robust psychometric properties, including its five-factor structure, internal consistency, and factorial invariance, support its use in research and applied psychology.

This study contributes to the growing body of research on emotion regulation and mental health by providing a culturally adapted tool for measuring behavioral strategies. Future investigations should continue to explore the BERQ-PA’s applications, ensuring its effectiveness in identifying risk and protective factors related to psychological well-being.

## Figures and Tables

**Table 1:** Sociodemographic Characteristics of the Sample (*n* = 403).

Sociodemographic Characteristics		Total Sample	Men	Women
		*n* (%)	(%)	*n* (%)
Educational Level	Secondary	18 (4.5)	8 (5.8)	10 (8.7)
	Technical superior	39 (9.6)	16 (11.6)	23 (9.6)
	University Superior	282 (70.0)	86 (62.3)	196 (74.0)
	Superior Postgraduate	64 (15.9)	28 (20.3)	36 (13.6)
Marital Status	Single	282 (70.0)	88 (63.9)	194 (73.2)
	Married/Cohabitant	109 (27.1)	46 (33.3)	53 (23.8)
	Divorced	3 (0.7)	2 (1.4)	1 (0.4)
	Widow(er)	9 (2.2)	2 (1.4)	7 (2.6)
Children	Yes	125 (31.0)	47 (34.1)	78 (29.4)
	No	278 (69.0)	91 (65.9)	187 (70.6)
Religion	Catholic	276 (68.5)	86 (62.3)	190 (71.7)
	Christian	30 (7.4)	12 (8.7)	18 (6.8)
	Other	49 (12.2)	20 (14.5)	29 10.9)
	Agnostic	48 (11.9)	20 (14.5)	28 (10.6)
Employment status	Work	265 (65.8)	100 (72.5)	165 (62.3)
	Does not work	138 (34.2)	38 (27.5)	100 (37.7)
Total		403 (100)	138 (34.2)	265 (65.8)

**Table 2:** Measures of Central Tendency, Dispersion, and Homogeneity Index of the BERQ-PA Items.

Items	*M*	*SD*	*g1*	*g2*	*r_it_*
BERQ1	3.34	1.11	−0.525	−0.244	0.361
BERQ 2	2.97	1.15	−0.001	−0.635	0.562
BERQ3	3.65	0.98	−0.603	0.166	0.506
BERQ 4	3.35	0.99	0.330	−0.378	0.355
BERQ 5	3.02	1.03	−0.119	−0.442	0.458
BERQ 6	3.46	0.97	−0.497	−0.058	0.471
BERQ 7	2.83	1.13	−0.051	−0.776	0.416
BERQ 8	3.48	0.96	−0.352	−0.042	0.647
BERQ 9	3.23	1.01	−0.344	−0.372	0.615
BERQ 10	2.21	1.04	0.468	−0.564	0.537
BERQ 11	3.56	0.95	−0.512	0.032	0.572
BERQ 12	2.34	1.18	0.540	−0.529	0.635
BERQ 13	3.59	0.99	−0.557	0.126	0.658
BERQ 14	3.31	1.00	−0.266	−0.318	0.586
BERQ 15	2.41	1.08	0.329	−0.665	0.670
BERQ 16	3.82	0.92	−0.559	0.075	0.507
BERQ 17	2.32	1.11	0.514	−0.426	0.697
BERQ 18	3.59	0.95	−0.395	−0.115	0.603
BERQ 19	3.22	1.01	−0.158	−0.448	0.661
BERQ 20	2.23	1.11	0.646	−0.321	0.567

*Note. M* = mean; *SD* = standard deviation; *g1* = skewness; *g2* = kurtosis; *r_it_* = corrected item-test correlation (homogeneity index).

**Table 3:** Goodness-of-fit values for the confirmatory factor analyses.

Model	S-B Scaled χ^2^ (df)	χ^2^/df	CFI	TLI	RMSEA (90% IC)	SRMR	ECVI
M1. Original five-factor model	366 *** (160)	2.29	0.947	0.937	0.050 (0.042–0.058)	0.071	1.047
M2. Alternative five-factor model (18 items)	299 *** (125)	2.39	0.959	0.950	0.048 (0.039–0.057)	0.069	0.829
M3. Two factors: (20 items—more adaptive and less adaptive strategies)	658 *** (169)	3.89	0.781	0.753	0.099 (0.092–0.106)	0.110	2.373
M4. Two factors: (18 items—more adaptive and less adaptive strategies)	574 *** (134)	4.28	0.805	0.778	0.101 (0.094–0.109)	0.110	1.975

*Note. df* = degrees of freedom; CFI = comparative fit index; TLI = Tucker–Lewis Index; RMSEA = root mean square error of approximation; SRMR = standard root mean square residuals; ECVI = expected cross-validation index (ECVI). *** *p* < 0.001.

**Table 4:** Factor Loadings of the BERQ-PA in CFA, Reliability, and Correlation between Sub-Scales.

Item	F1	F2	F3	F4	F5
BERQ6	0.651				
BERQ 11	0.796				
BERQ 16	0.560				
BERQ 2		0.602			
BERQ 7		0.557			
BERQ 12		0.763			
BERQ 17		0.814			
BERQ 3			0.518		
BERQ 8			0.786		
BERQ 13			0.792		
BERQ 18			0.695		
BERQ 9				0.721	
BERQ 14				0.755	
BERQ 19				0.780	
BERQ 5					0.555
BERQ 10					0.648
BERQ 15					0.720
BERQ 20					0.734
Internal Consistency					
α IC 95%	0.704 [0.65:0.75]	0.772 [0.73:0.81]	0.793 [0.76:0.82]	0.796 [0.76:0.83]	0.759 [0.72:0.80]
ω IC 95%	0.718 [0.67:0.77]	0.782 [0.75:.82]	0.797 [0.76:0.83]	0.797 [0.76:0.83]	0.768 [0.73:0.80]
α_ordinal_	0.754	0.808	0.830	0.829	0.800
ω_ordinal_	0.775	0.808	0.840	0.834	0.838
Factor correlation					
F1	-				
F2	0.168 **	-			
F3	0.440 ***	−0.172 ***	-		
F4	0.321 ***	−0.053	0.596 ***	-	
F5	0.454 **	0.587 ***	−0.135 ***	0.006	-
*M* (*DE*)	10.8 (2.3)	10.5 (3.5)	14.3 (3.1)	9.7 (2.5)	9.9 (3.2)

*Note.* F1 = seeking distraction; F2 = withdrawal; F3 = actively approaching; F4 = seeking social support; F5 = ignoring. ** *p* < 0.01; *** *p* < 0.001.

**Table 5:** Pearson and Partial Correlations between the BERQ-PA Subscales and GHQ-28 Dimensions.

Subscales	Somatic Symptoms		Anxiety		Social Dysfunction		Depression	
	*r*	*r_p_*	*r*	*r_p_*	*r*	*r_p_*	*r*	*r_p_*
1. Seeking Distraction	−0.112 *	−0.155 **	−0.09	−0.140 **	−0.200 **	−0.165 **	−0.115 *	−0.181 ***
2. Withdrawal	0.199 **	0.174 ***	0.210 **	0.164 **	0.196 **	0.171 **	0.269 **	0.193 ***
3. Actively Approaching	−0.072	−0.011	−0.089	−0.041	−0.245 **	−0.121 *	−0.149 **	−0.051
4. Seeking Social Support	0.023	0.072	0.042	0.100 *	−0.144 **	−0.025	−0.022	0.057
5. Ignoring	0.098 *	0.060	0.138 **	0.089	0.073	0.033	0.213 **	0.158 *

*Note. r_p_* = Partial Correlation Coefficient. * *p* < 0.05; ** *p* < 0.01; *** *p* < 0.001.

**Table 6:** Correlations between the BERQ-PA subscales and the ERQ subscales.

ERQ Subscales	BERQ Subscales				
	Seeking Distraction	Withdrawal	Actively Approaching	Seeking Social Support	Ignoring
Cognitive Reappraisal	0.265 **	−0.136 **	0.281 **	0.127 *	0.054
Expressive Suppression	0.040	0.278 **	−0.176 **	−0.174 **	0.346 **

* *p* < 0.05; ** *p* < 0.01.

**Table 7:** Goodness of Fit Indices of the Models Evaluated in Factor Invariance Analysis.

Model	*χ*^2^ (*df*)	TLI	CFI	RMSEA (90% IC)	SRMR	∆*χ*^2^ (*df*)	∆CFI	∆RMSEA	∆SRMR
MI 1. Configurational Invariance	289.33 * (250)	0.983	0.986	0.028 (0.005–0.041)	0.075				
MI 2. Metric Invariance	321.12 * (263)	0.976	0.979	0.033 (0.018–0.045)	0.079	31.79 (13)	0.007	0.005	0.005
MI 3. Scalar Invariance	323.85 * (276)	0.981	0.983	0.029 (0.011–0.042)	0.076	2.73 (13)	0.004	0.004	0.003
MI 4. Strict Invariance	335.66 * (294)	0.985	0.985	0.027 (0.003–0.039)	0.077	11.81 (18)	0.002	0.002	0.001

* *p* < 0.05.

## Data Availability

The original data presented in the study are openly available on https://osf.io/t4kna/?view_only=852e4af94fb74df8be5f885b8024d051 (accessed on 5 November 2024).

## Supplementary Materials

The following supporting information can be downloaded at https://www.mdpi.com/article/10.3390/bs15020224/s1. File S1: Peruvian and original version of the BERQ.
