# Supplementary material for: Psychometric Properties of the Behavioral–Emotional Regulation Questionnaire in Peruvian Adults (BERQ-PA)
Source: Behav Sci (Basel). 2025 Feb 16;15(2):224. doi: 10.3390/bs15020224 (PMC11851976; doi:10.3390/bs15020224)
Supplement: Supplementary file 1 [file behavsci-15-00224-s001.zip › behavsci-3319089-supplementary.pdf]

Supplementary material

S1. Peruvian and original versions of the BERQ

	Peruvian Version (Domínguez-Lara et al., 2022)	Original Version (Kraaij & Garnefski, 2019)
Title	Cuestionario de Regulación Conductual de las Emociones	Behavioral Emotion Regulation Questionnaire (BERQ)
Instructions	Todos nos enfrentamos a situaciones negativas o desagradables de vez en cuando y todos respondemos a ellas de nuestra manera. Con las siguientes preguntas, se le pide que indique qué es lo que generalmente hace cuando experimenta una situación negativa o desagradable. Lea las siguientes oraciones e indique la frecuencia con que se aplican a usted. Marque con una "X" la respuesta que mejor lo describa.	How do you deal with what happens in your life? Everyone gets confronted with negative or unpleasant events now and then and everyone responds to them in his or her own way. By the following questions you are asked to indicate what you generally do, when you experience negative or unpleasant events. Read the sentences below and indicate how often they apply to you. You can do so by circling the answer which best describes you.
Rating Scale	CN = Casi nunca o nunca<br>AV= A veces<br>N= Normalmente<br>F = Frecuentemente<br>CS = Casi siempre o siempre	(almost) Never<br>Sometimes<br>Regularly<br>Often<br>(Almost) Always
Item 1 (Seeking Distraction)	Me dedico a otras actividades que no estén relacionada a lo que sucede	I engage in other, unrelated activities
Item 2 (Withdrawal)	Evito a las personas	I avoid other people
Item 3 (Actively Approaching)	Trato de hacer algo al respecto	I try to do something about it
Item 4 (Seeking Social Support)	Busco que alguien me consuele	I look for someone to comfort me

	**Peruvian Version (Domínguez-Lara et al., 2022)**	**Original Version (Kraaij & Garnefski, 2019)**
Item 5 (Ignoring)	Sigo adelante y pretendo que no pasó nada	I move on and pretend that nothing happened
Item 6 (Seeking Distraction)	Dejo de lado mis preocupaciones haciendo otra cosa	I set my worries aside by doing something else
Item 7 (Withdrawal)	Me retiro	I withdraw
Item 8 (Actively Approaching)	Me pongo a trabajar sobre ello	I get to work on it
Item 9 (Seeking Social Support)	Pido consejo a alguien	I ask someone for advice
Item 10 (Ignoring)	Lo reprimo y pretendo que nunca sucedió	I repress it and pretend it never happened
Item 11 (Seeking Distraction)	Hago otras cosas para distraerme	I do other things to distract myself
Item 12 (Withdrawal)	Me aíslo	I isolate myself
Item 13 (Passion Criteria)	Tomo medidas para enfrentarlo	I take action to deal with it
Item 14 (Seeking Social Support)	Comparto mis sentimientos con alguien	I share my feelings with someone
Item 15 (Ignoring)	Me comporto como si nada estuviera pasando	I behave as if nothing is going on
Item 16 (Seeking Distraction)	Me dedico a una actividad que me haga sentir bien	I engage in an activity which makes me feel good
Item 17 (Withdrawal)	Me alejo de los demás	I close myself off to others
Item 18 (Actively Approaching)	Hago lo que sea necesario para afrontarlo	I do whatever is required to deal with it
Item 19 (Seeking Social Support)	Busco alguien que me pueda apoyar	I look for someone who can support me
Item 20 (Ignoring)	Bloqueo lo que está sucediendo	I block it out
